# Comparison of Outcome Data for Shelter Dogs and Cats in the Czech Republic

**DOI:** 10.3390/ani9090595

**Published:** 2019-08-22

**Authors:** Veronika Vojtkovská, Eva Voslářová, Vladimír Večerek

**Affiliations:** Department of Animal Protection, Welfare and Behavior, Faculty of Veterinary Hygiene and Ecology, University of Veterinary and Pharmaceutical Sciences Brno; 612 42 Brno, Czech Republic

**Keywords:** adoption, canine, feline, length of stay, animal rescue

## Abstract

**Simple Summary:**

Knowing the factors that influence animal adoption is a key step towards making changes in shelters-the goal is to increase the adoption rates and to reduce the time spent by animals in shelters. The aim of the study was to compare the length of stay of dogs and cats in the selected shelters in the Czech Republic. A significantly different length of stay of dogs and cats in the shelters before adoption was found. Cats seem to have a worse chance of being adopted in comparison with shelter dogs.

**Abstract:**

Animal protection, which also comprises the subject of abandoned and stray animals, has become a pressing and widely discussed topic. The aim of this study was to compare dogs and cats from two shelters in a selected region of the Czech Republic, based on factors that affect the length of stay. The following factors were analyzed: outcome, sex, age, and purebred status. A total of 419 cats and 2580 dogs housed in the monitored shelters from 2013 to 2016 were included in the analysis. The results show that dogs (n = 1343; 52.1%) are returned to their owners significantly more often (*p* < 0.001) than cats (n = 10; 2.4%). Dogs stayed in the shelter significantly (*p* < 0.001) less time than cats regardless of the outcome (the median length of stay of dogs were 3 days, while that of cats was 51 days). Also the length of stay in the shelter until adoption is shorter in dogs than in cats (dogs: median 27 days; cats: median 53 days). Median length of stay tended to increase with the increasing age in both species. Monitored age categories of dogs and cats differed significantly (*p* < 0.05) in their median length of stay (LOS) until adoption. We found that purebred status does not affect the length of stay in the shelter until adoption, either in dogs or in cats. Overall, our results suggest that dogs are preferred over cats in the Czech Republic. Shelter operators should take into account this aspect affecting animal adoption. By targeted efforts and education of public, it is possible to mitigate the negative effects of favoring a certain category of animals over others.

## 1. Introduction

The issue of abandoned and stray animals is a widely discussed topic of the present. Cats and dogs come to the shelter for four most common reasons: 1. they were caught as stray animals; 2. they were brought to the shelter by an owner who could no longer take care of them or did not want to; 3. they were returned to the shelter after adoption or 4. placed in a shelter after being confiscated from owners who violated the animal welfare legislation [1,2]. The importance of shelters lies in the tasks performed by these facilities. Since the health status of the admitted animals is usually unknown [3], shelters fulfil a protective role (thus maintaining public health and safety). Stray animals can pose a threat to the public in terms of zoonotic diseases. By trapping them, shelters reduce a risk of dangerous encounters between people and animals. Public education and edification are other essential tasks. And last but not least, shelters are trying to provide proper living conditions for ownerless animals and to ensure their adequate care.

In the Czech Republic, efforts to protect stray and abandoned animals are included in the individual legal regulations which include conditions for the establishment and management of shelters. An important fact is that legislation does not allow animals to be killed without justification; exceeding the capacity of the shelter, lack of adoptions or high age of the animal are not the reasons for legal euthanasia in the Czech Republic, as is the case for example in the United States [4], Australia [5], or Japan [6]. Knowing the factors that influence animal adoption is thus a key step towards making changes in shelters - the aim is to increase the adoption rates and to reduce the length of stay of animals in shelters.

The factors influencing adoption rates and the length of stay of dogs in the shelters were dealt with by a number of foreign authors [2,7,8,9,10,11,12]; the same is true for cats [13,14,15,16,17,18,19,20]. The tendency to compare the factors influencing adoption and the length of stay in the shelter for both species has been recorded [21,22,23], however, the results of a statistical analysis of interspecific comparisons within the same region and the observed residence time of animals in shelters have not been published yet.

Studies that deal with the factors affecting adoptions of dogs and cats may differ in their results depending on the country. The differences are determined by status and relationship to dogs and cats in the country. The number of dogs kept in the Czech Republic is significantly higher than the number of cats kept (the number of dogs kept in the Czech Republic in 2017 was about 2,150,000 [24], while the amount of cats was around 1,100,000 [25]; it is estimated that almost one half of all Czech households own one or more dogs [26]). Contrary, in Germany, France, Italy, the Netherlands, or the US, a cat is kept as a pet more often than a dog [24,25]. The opposite trend can be seen in Australia - about 38% of all households own a dog, while only 29% have a cat [27]. The reason for the popularity of cats may lie in the seemingly easier care (unlike a dog, a cat does not require walking). The position of cats in the Czech Republic is still partly based on the historical image of the rural landscape - the cat was primarily attributed the role of a guardian of the dwelling from rodents and its value was not perceived as high. Unlike dogs, the trend of owning a cat as a companion animal does not have a long tradition in the Czech Republic. No study has been published to investigate why dogs are popular in the Czech Republic; however, it seems to be necessary to look into the past to answer the question. The popularity of dogs is probably based on the strong relationship of a dog and a man, which has been shaped across history. In the past, people made their living mainly by cattle breeding. Dogs helped people with herding and guarding cattle but they also accompanied shepherds. Other than the dog, shepherds usually had no companionship while working; therefore the strong human-animal bond could be developed. Nowadays, shepherd dogs are not used as commonly as in the past. They can be usually seen on farms and in the countryside [28]. In the 16th to 19th centuries, Czech land was the main hunting district of the Austro-Hungarian Empire. In those times, hunting dogs were widely used to help people to hunt. Breeding of this type of dogs has a long tradition in the Czech Republic [29]. This may be another reason for the overall popularity of dogs in this country.

The perception of differences in dog and cat preferences is an important aspect of successful adoption; shelters can by their activity contribute to awareness of species that seem less popular in society and thus increase their adoption potential.

The efforts of Czech authors to describe the factors affecting the length of stay and adoption rates of animals in shelters separately in dogs and in cats were carried out through several studies. The effect of sex, age, and coat color on the admission of cats to the shelter and the length of their stay in the shelter until adoption in the Czech Republic was studied by Kubesova et al., [30] and Voslarova et al. [31]. The authors monitored three cat shelters in the Czech Republic. Originally, 2170 cats were admitted to the shelters, of which 1407 (65%) were adopted. The authors found a relatively long median length of stay (LOS) of cats until adoption (69 days). The total median LOS of cats (regardless of the outcome) was 45 days. Only 2% of cats were reclaimed from the shelters by their original owners. Significantly more female cats were adopted than male cats; however, the median time until adoption did not differ significantly between males and females. Significantly greater numbers of female cats (56%) than male cats were admitted. Most cats (60%) were admitted if they were aged younger than six months. The age of cats affected the time until adoption. Geriatric cats had the greatest (median LOS more than 120 days) and adult cats the shortest LOS (less than 60 days). The median LOS of dark-colored cats was significantly greater than that of cats with light or medium shade of coat color. Other Czech authors [32] investigated the mortality rate and the length of stay of cats in shelters until death or euthanasia. Death was the ultimate outcome for 33% of cats admitted in the shelters (22% of cats died and 11% were euthanized). The majority of mortalities occurred shortly after admission. The factors (sex, age, and size) affecting the length of time for which dogs stay in shelters were studied by Zak et al. [3]. The authors monitored three dog shelters in which a total of 3875 dogs were housed. Out of these, 41.7% of dogs were reclaimed by their owners and 58.3% of dogs were regarded as being abandoned by their owners and were offered for adoption. The median LOS of all abandoned dogs was 23 days. The median LOS of all lost dogs was 1 day. Abandoned male dogs remained in the shelters for significantly longer (median 27 days) than abandoned females (median 21 days). Young abandoned dogs up to the age of one year had the shortest LOS (median 19 days), whereas older dogs had the longest LOS (53.5 days). Abandoned dogs over 65 cm at the withers and dogs up to 35 cm had the shortest LOS in the shelters. Research of the influence of the breed as another factor was carried out by Voslarova et al. [33]. In abandoned dogs, purebred status had a significant effect on the time the dog stayed in the shelter until adoption. The median time until the adoption of crossbred dogs was 27 days, whereas that of purebred dogs was 19 days. The factors affecting adoption of dogs were also investigated in an older study by Nemcova and Novak [34]. The most frequently adopted dogs from the shelters were those aged 2–4 months. Mixed breed dogs were sheltered and also adopted more often than dogs with the exterior signs of a specific breed.

A study comparing dogs and cats within one region and the same reference period is still missing. Based on the results of previous studies, cats seem to have a worse chance of being adopted in comparison with shelter dogs. In this study, we decided to test this hypothesis. Knowing the differences between LOS of dogs and cats in the shelters and the factors affecting LOS can help to understand the perception of both species by potential adopters. By investigating the differences in factors, we may help shelter personnel to establish effective procedures of seeking new homes, particularly for animals which seem to be less likely to be adopted and thus decrease their LOS. This is very important in no-kill shelters where the LOS is not limited. By decreasing LOS, the welfare of an animal can be improved. The aim of the present study was therefore to compare the population of dogs and cats from selected shelters in terms of the length of stay. The impact of four factors was assessed and compared: outcome, sex, age, and purebred status.

## 2. Materials and Methods

Records on shelter dogs and cats were collected from two shelters situated in one region of the Czech Republic. The subject of this retrospective study was all dogs and cats housed in the shelters from 1 January, 2013 to 31 December, 2016. The capacity of the mixed shelter (dogs and cats in one facility) was 80 dogs and 10 cats; the capacity of the second evaluated facility (solely feline shelter) was 30 cats. Both shelters were “no-kill” shelters by law. In both shelters, incoming animals were firstly housed in a quarantine area, which was separated from the accommodation for healthy animals. The length of the quarantine period depended on the health condition of the animal; it usually lasted 14 days. During the quarantine period, the dogs and cats were treated and basic veterinary procedures were performed if the animal’s health state enabled this to occur. The animals were microchipped, dewormed, and the antiparasitic treatment of internal and external parasites was performed. Dogs and cats were also vaccinated against the most common infectious diseases. Rabies vaccination was performed in both dogs and cats, however, in the Czech Republic mandatory vaccination only applies to domestic dogs. It was a routine practice in the monitored shelters to desex cats. In contrast, routine neutering of dogs in the selected shelter was not performed. After the quarantine period, dogs were housed in double compartment kennels individually, while cats were placed in group rooms in both monitored shelters. Cats in the mixed shelter were kept out of sight of dogs in a separated part of the facility. Cats and dogs showing any signs of infectious disease were moved immediately to the isolation area. The shelter caregiver monitored the animals’ health and behavior daily; however, no official health or behavioral assessment tool was used to evaluate the animals. In both shelters, none of the animals were euthanized for behavioral issues.

Data for evaluation were collected from 2580 dogs and 419 cats. Data included intake date, information about the animals’ breed, approximate or known age, sex, and the date when the stay in the shelter was terminated. The records also contained the information on how the stay in the shelter was terminated (adoption, unassisted death, euthanasia for health reasons or return to the original owner). In the case of cats, cases of releasing the animal to the capture location were also recorded. The capture of feral cats was performed due to veterinary treatment (neutering, vaccination, and treatment against internal and external parasites).

In order to determine the effects of monitored factors on the length of stay of animals in the shelter, the LOS (the length of stay of the animal in the shelter) was calculated in days, which represented the difference between the intake date and the date when the stay in the shelter was terminated. Median LOS was calculated for dogs and cats in each way of the outcome (adoption, unassisted death, euthanasia for health reasons and return to the original owner). Total LOS represents the total time (median) a dog or a cat stayed in a shelter regardless of the final outcome.

The monitored canine and feline population was assessed in terms of how the stay in the shelter was terminated. In both species’ rates of adoption, unassisted death, euthanasia, or returning to the original owner were calculated. In cats, numbers of animals released to the capture location were also counted. Cats and dogs that remained in the shelters after 31 December, 2016, were not included in the analysis. Based on the known or estimated age, the animals from both species were divided into 4 categories: dogs and cats from 0 to 6 months inclusive; dogs and cats older than 6 months of age up to and including 1 year; dogs and cats older than 1 to 6 years inclusive, and finally dogs and cats older than 6 years. The breeds of dogs and cats were determined by shelter workers based on the exterior signs. Two categories of animals were created for evaluation purposes - a group of animals that had characteristics specific to a particular breed (purebred animals), and a group which did not show signs of belonging to any breed (crossbreds).

The results were analyzed using the statistical package Unistat 5.6. (Unistat Ltd., London, UK). Five independent variables were created based on the data obtained from shelters: animal species (2 levels: dog, cat), way of outcome (4 levels: adoption, unassisted death, euthanasia, returning to original owner), sex (2 levels: male, female), age (4 levels: x ≤ 6 months, 6 < x ≤ 12 months, 1 < x ≤ 6 years, x > 6 years; x = age of an animal), purebred status (2 levels: purebreds, crossbreds). The effect of these variables on LOS in the shelters was analyzed. For LOS, normality was checked using the Kolmogorov-Smirnov test [35]. The data were not distributed normally, so we used non-parametric methods for statistical analyses. Firstly, the median for LOS was calculated for each level of variables in cats and dogs. The effect of 2-level variables was analyzed using a two-tailed Mann-Whitney U test. The effect of variables with several levels was analyzed by Kruskal-Wallis ANOVA [35]. Subsequently, when the effect of the variable was significant, a non-parametric Tukey-type test was used as a post-hoc test for pairwise comparisons. We also calculated actual and relative frequencies of cats and dogs in selected categories. Frequencies were compared on the basis of chi-square analysis of 2 × 2 and k × m contingency tables [35]. A *p-*value < 0.05 was considered significant.

## 3. Results

The total LOS (length of stay in the shelters regardless of the way of outcome) differed significantly between cats and dogs (*p* < 0.001). The total median LOS of cats was 51 days (mean total LOS = 107 days, range = 0–1534 days) while that of dogs was only 3 days (mean total LOS = 34 days, range = 0–2564 days).

The type of final outcome after being the shelter affects the LOS in dogs and cats. Table 1 shows the numbers of animals according to their outcome (adoption, unassisted death, euthanasia, return to an owner and release to the capture location) and the median LOS. The numbers of adopted dogs (n = 1073, 41.6%) and cats (n = 367, 87.6%) differed significantly (*p* < 0.001). Cats stayed in the shelters significantly longer until adoption (*p* < 0.001) compared to dogs (adopted cat median LOS = 53 days, mean LOS = 102 days, range = 3–1534 days; adopted dog median LOS = 27 days, mean LOS = 62 days, range = 0–1561 days). A significant difference between the number of dogs (n = 56, 2.2%) and cats (n = 17, 4.1%) that died in the shelter was found (*p* < 0.05). While the median LOS of dog or a cat until unassisted death did not differ significantly (*p* > 0.05) (canine median LOS = 13 days, canine mean LOS = 76 days, range = 1–1707 days; feline median LOS = 25 days, feline mean LOS = 56 days, range = 2–298 days), the length of stay in the shelter until euthanasia was different. Dogs stayed in the shelter before euthanasia for significantly longer (*p* < 0.001) than cats (canine median LOS = 79 days, canine mean LOS = 411 days, range = 3–2564 days; feline median LOS = 7 days, feline mean LOS = 7 days, range = 0–14 days). The difference between the number of euthanized dogs (n = 32, 1.2%) and cats (n = 2, 0.5%) was insignificant (*p* > 0.05). Lost cats stayed in the shelter until returning to their original owners significantly longer (*p* < 0.001) than lost dogs (feline median LOS = 4 days, feline mean LOS = 13 days, range = 1–98 days; canine median LOS = 0 days, canine mean LOS = 2 days, range = 0–161 days). The number of reclaimed dogs (n = 1343, 52.1%) and cats (n = 10, 2.4%) differed significantly (*p* < 0.001). No significant difference was found (*p* > 0.05) when comparing the numbers of unadopted dogs (n = 76, 2.9%) and cats (n = 14, 3.3%). In the case of cats, a number of released animals to the capture location was also recorded (n = 9, 2.1%). The median LOS of these cats was 40 days (mean LOS = 106 days, range = 7–737 days).

**Figure 1:**
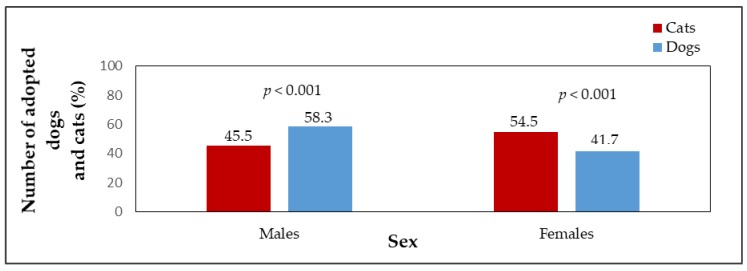
The percentage of adopted dogs (n = 1073) and cats (n = 367) in sex categories.

**Figure 2:**
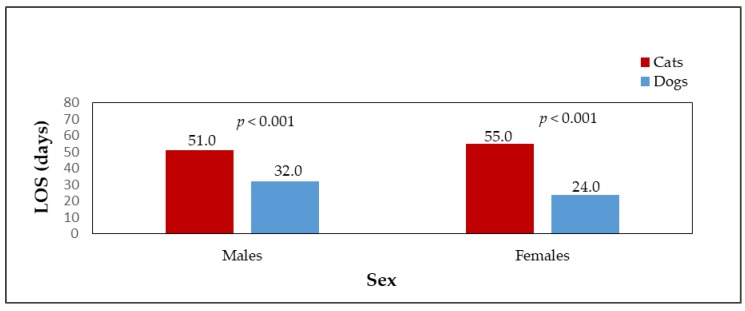
The median LOS of adopted dogs (n = 1073) and cats (n = 367) in sex categories.

**Figure 3:**
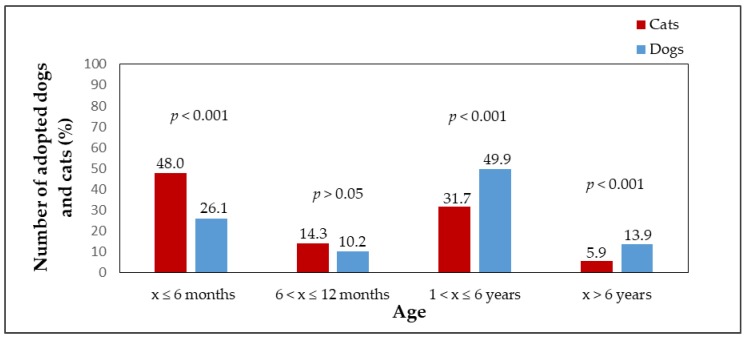
The percentage of adopted dogs (n = 1051) and cats (n = 356) in four age categories, x = age of an animal.

**Figure 4:**
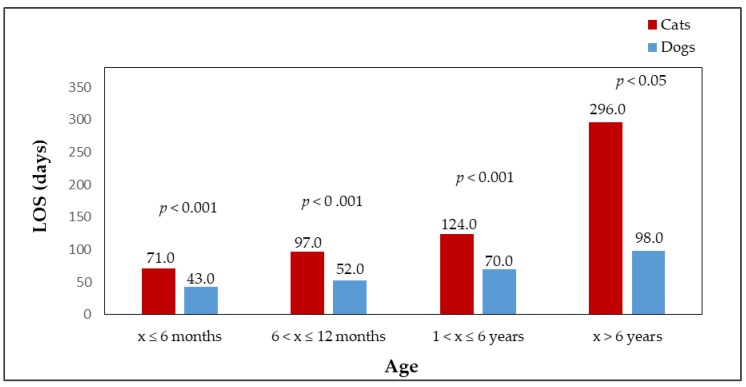
The median LOS of adopted dogs (n = 1051) and cats (n = 356) in four age categories; x = age of an animal.

Adopted dogs and cats were also compared in terms of sex. The percentage of females and males adopted from shelters is shown in Figure 1. A significant difference (*p* < 0.001) was found between the number of adopted male dogs (n = 626, 58.3%) and male cats (n = 167, 45.5%) and female dogs (n = 447, 41.7%) and female cats (n = 200, 54.5%). Figure 2 shows the median LOS of males and females in the shelter until adoption. Male cats stayed in the shelter for a slightly shorter time than female cats (*p* > 0.05, male cats median LOS = 51 days, male cats mean LOS = 89 days, range = 3–1090 days; female cats median LOS = 55 days, female cats mean LOS = 113 days, range = 4–1534 days). Female dogs stayed in shelters for a significantly shorter time than male dogs (*p* < 0.001, female dogs median LOS = 24 days, female dogs mean LOS = 46 days, range = 0–695 days; male dogs median LOS = 32 days, male dogs mean LOS = 73 days, range = 0–1561 days). The differences in the length of stay in the shelter until adoption were statistically significant among the sexes of the species (*p* < 0.001). Female cats stayed in the shelter until adoption for significantly longer times (median LOS = 55 days) than female dogs (median LOS = 24 days); male dogs were adopted significantly faster (median LOS = 32 days) than male cats (median LOS = 51 days). Figure 3 shows the percentages of adopted dogs and cats across age categories. A significant difference was found between the numbers of adopted dogs (n = 274, 26.1%) and cats (n = 171, 48%) in the youngest age category (*p* < 0.001). Also the numbers of the oldest dogs (n = 146, 13.9%) and cats (n = 21, 5.9%), and adult dogs (n = 524, 49.9%) and cats (n = 113, 31.7%) differed significantly (*p* < 0.001). An insignificant result was found when comparing adopted dogs (n = 107, 10.2%) and cats (n = 51, 14.3%) in the age category of animals from 6 to 12 months (*p* < 0.05). The median LOS of dogs and cats until adoption was prolonged with the increasing age of the animals (Figure 4).

The shortest stay was recorded in the youngest dogs and cats (from 0 to 6 months of age including), i.e., cats: median LOS = 43 days, mean LOS = 71 days, range = 3–448 days; dogs: median LOS = 21 days, mean LOS = 26 days, range = 0–331 days. Old animals (over 6 years of age) remained the longest in shelters among all evaluated categories (cats: median LOS = 98 days, mean LOS = 296 days, range = 10–1293 days; dogs: median LOS = 53 days, mean LOS = 93 days, range = 0–1062 days). There was no significant difference (*p* > 0.05) between the median LOS of oldest category in dogs (age over 6 years) and the youngest category in cats (from 0 to 6 months including). A similar result was found when comparing the median LOS of category of young cats (from 6 months to 1 year including) and the category of the oldest dogs (*p* = 0.8224). In all other evaluated combinations of comparisons between cats and dogs, a significant difference (*p* < 0.05) was found. By assessing the median LOS in dogs and cats within the species, age was found to affect the length of stay until adoption in both dogs and cats.

**Figure 5:**
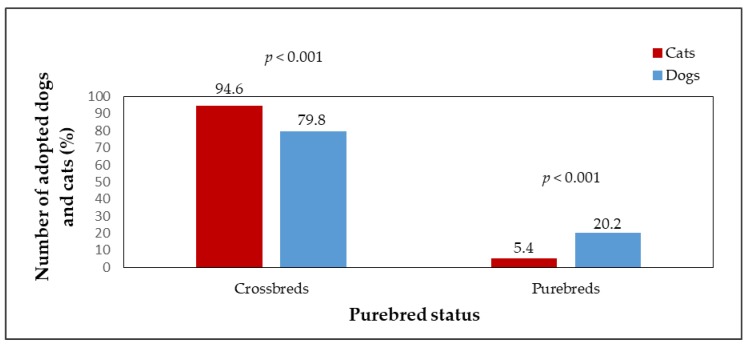
The percentage of adopted dogs (n = 1072) and cats (n = 367) as affected by purebred status.

**Figure 6:**
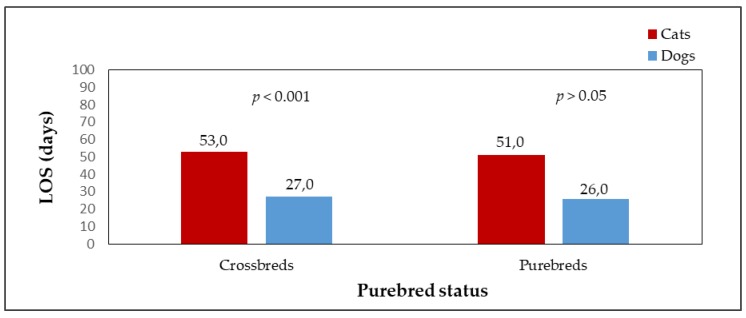
The median LOS of dogs (n = 1071) and cats (n = 367) as affected by purebred status.

Adopted dogs and cats were also evaluated in terms of their purebred status. The numbers of adopted purebred and crossbred animals are shown in Figure 5. The numbers of adopted crossbred dogs (n = 855, 79.8%) and crossbred cats (n = 347, 94.6%), and purebred dogs (n= 216, 20.2%) and purebred cats (n = 20, 5.4%) differed significantly (*p* < 0.001). The median LOS of purebred cats and dogs in the shelter until adoption (Figure 6) was shorter than that of crossbreds. A significant difference was found when comparing the median LOS of crossbred dogs and crossbred cats (*p* < 0.001; crossbred dogs median LOS = 27 days, crossbred dogs mean LOS = 62 days, range = 0–695 days; crossbred cats median LOS = 53 days, crossbred cats mean LOS = 105 days, range = 3–1534 days). Crossbred dogs stayed in the shelter for a significantly shorter time than crossbred cats. In the case of purebred dogs and cats, the median LOS in the shelter did not differ significantly (p > 0.05; purebred dogs median LOS = 26 days, purebred dogs mean LOS = 62 days, range = 0–967 days; purebred cats median LOS = 51 days, purebred cats mean LOS = 57 days, range = 10–283 days).; however The difference in median LOS between purebred and crossbred cats was insignificant (*p* > 0.05). The same result was found in dogs (median LOS between purebred and crossbred dogs did not differ significantly).

## 4. Discussion

According to our results, dogs stayed in the Czech shelters for significantly less time than cats, regardless of the outcome. A similar result was observed in the case of the assessment of LOS until adoption - dogs were adopted from the shelter in half the adoption time for cats. The short overall time of stay in the shelter in dogs was caused by the fact that more than a half of the dogs (52.1%) admitted to the monitored shelter were almost immediately returned to their original owners. According to Zak et al., [3] dogs in the Czech Republic stay in a shelter until adoption for 23 days, which is a result similar to our findings, while according to Kubesova et al., [30] cats from Czech shelters are rehomed in 69 days (median). When comparing the studies of Czech authors who dealt with different species individually, the same phenomenon was observed as in our study - dogs stay in shelters for shorter times than cats. In addition, several foreign authors monitored the length of stay of dogs and cats in a shelter until adoption and the preference of a certain species to others and the results of the studies varied depending on the country. While in Sweden, a cat from a shelter is rehomed after 88 days (median) [36], in the UK the time until adoption is shorter at only 30 days (median). In the UK, a similar period of stay in the shelter dogs until adoption was assessed by Diesel et al. [37]. Authors found that the median length of stay of an adopted dog was 28 days [37]. American authors mention 61 days (median) in case of cats [17]; dogs were adopted after 34 days (median) [38]. The difference in the length of stay in a shelter until adoption between dogs and cats in our study may be due to the fact that in the Czech Republic dogs, rather than cats, are the preferred companion animal [26]. In assessing the results, it is also necessary to build on historical socio-cultural conventions that reflect the relationship between a person and an animal in the Czech Republic. Unfortunately, it is still possible to hear the common opinion that a cat is an animal that primarily serves to catch rodents and cannot be as a close a companion to a human being as a dog. This view is also supported by the individualistic nature of the cat, which is often misinterpreted by the general public as being false and insidious. However, to our knowledge, no studies have been published in the Czech Republic to confirm this phenomenon.

According to our results, the shortest time of stay occurs for cats and dogs that have been lost by their original owners. The result is consistent with the findings of the study by Voslarova et al., [33] who found that male dogs were returned to the original owner after one day in the shelter and females even on the day of admission (median 0 days). Unlike dogs, which made up 41.7% of returned animals [33], the percentage of returned cats is low - our findings show only 2.4% cases of cats being returned to their original owner. Similar results were obtained by Kogan et al., [16] who recorded a 2% rate and Albertsen et al., [5] who recorded a 3% rate. The differences between cats and dogs in the length of stay until returning to owners (as well as the rate of returns) need to be assessed on the basis of the situation with the animal identification in the Czech Republic. Dogs tend to be identified by their owners more often than cats e.g., by a tattoo, chip, collar with a mark, etc., and thus their subsequent identification by their owners is easier. It is not mandatory for people to microchip their dogs in the Czech Republic, however, it was found that 48.2% of dogs admitted to shelters in the Czech Republic were somehow identified [39]. In the case of lost cats, the owners are not accustomed to looking for their pets in shelters, as is the common practice for dogs. Contacting a shelter to see if a cat is lost is one of the last options the owners pursue in order to find their pet. Lost cats stay in shelters longer-actually until the owner finds out that their cat has been caught and is in a shelter. When the animal enters the animal shelter, the facility contacts the owner on the basis of the animal’s identification (if the animal is identified). However, a problem occurs when the animal is microchipped, but the owner has not registered the chip number in the records. In the Czech Republic, the veterinarian who applied the microchip is not obliged to register the animal; this must be done by the owner. The veterinarian only registers the animal in the register if they have also been issued with a pet passport. If the animal that does not have a pet passport is not registered, the shelter does not have any way to find the owner.

In addition to adoption and returning the animal to the original owner, death and euthanasia are other ways for the stay in the shelter to be terminated. While the number of cats and dogs put to death in selected shelters did not differ significantly, the difference in the number of dogs and cats that died in the shelters was significant. A higher percentage of cats died in shelters than dogs. In general, however, according to the results, the mortality and euthanasia rates in the monitored shelters were very low (the number of dead and euthanized cats and dogs was below 5%), which may indicate that the management of the quarantine system and the veterinary care was appropriate in the monitored facilities. A number of studies have been published to investigate the mortality rates in shelters [5,22,40,41]; however, it is important to note that only some results can really be taken into account for the comparison. The Czech Republic is among the countries that do not allow animals in shelters to be killed because of the shelter’s capacity or any reasons other than health reasons. Reasons for legal euthanasia under the Animal Protection Act include weakness, incurable illness, severe injuries, genetic and birth defects, general exhaustion, or aging of the animal in cases when the prolonged survival of the animal would mean permanent suffering. Similarly to the Czech Republic, Brazil since 2008 has been included among countries that do not permit euthanasia for reasons other than health; however, Brazilian studies mention a mortality rate of up to 40% in dogs and cats [42], which is a very different result compared to our findings. Also, the authors of another Czech study reported a high mortality rate in shelter cats. They found that 33% of all cats admitted to the shelter died or were euthanized, while the number of cats that died was almost twice the number of cats that were put to death [32].

In our study, the length of stay in the shelter until euthanasia was significantly shorter in cats than that observed in case of euthanized dogs. In general, a bad health condition is one of the most common reasons for euthanasia of animals in shelters [43]. The risk of death decreases with the increasing length of time the animal stays in the shelter [41]. After admission, there is an effort to eliminate the life-threatening conditions in which the animal is often taken to the facility. Cave et al., [43] found a number of cats and dogs being put to death on the very first day, even on the day of their capture. In the facilities monitored in our study, in the case of cats, the tendency to euthanize animals on the first day of stay is reflected in the results - the median residence time of the euthanized cats was one week. In dogs, the situation is different not many dogs in critical condition were taken to the monitored shelters; shelter employees chose euthanasia as the last option to alleviate the suffering of long-term ill dogs. As we have already mentioned, in the Czech Republic it is forbidden to euthanize animals for other than health reasons under the Animal Protection Act. However, if it is found that an animal’s behavioral problem has a health-related cause, it is possible to put the animal to death. However, shelters usually do not have sufficient financial resources for an animal to undergo a proper examination to reveal the health cause of its problematic behavior. In this case, the animal in question remains in the shelter, although its behavior may be unacceptable for potential adopters. In the shelters that we monitored, none of the animals was euthanized due to a behavioral problem. Although a large number of animals with disrupted medical conditions end up into shelters, the shelters themselves can also play an important role in increasing the incidence of diseases, as the infectious pressure exerted on the animal in the shelter environment may rise as a result of a prolonged stay [44]. Comparing the length of stay of animals in a shelter until euthanasia with a foreign study, Brazilian authors found a rate of 9 days (median) spent in a shelter until euthanasia for both dogs and cats (the study evaluated both species together) [42]. According to Czech authors Vecerek et al., [32] the length of stay of cats in shelters until death ranged from 16 to 26 days (median) depending on the age and sex of the animal; the shortest time until euthanasia was found in the cat category over 50 months of age (median 0 days). Young animals are most likely to be the subject of death and euthanasia - the key fact is that the immune system of this category of animals is not yet fully developed, which may mean an increased susceptibility to infectious diseases [32]. In terms of mortality and euthanasia, senior animals are another risk group [41].

The last monitored means of terminating the stay in selected shelters was in the case of cats returning to the place of capture after veterinary treatment. The median residence time of cats with this purpose of stay in a shelter was 40 days; this result cannot be compared with the canine population (in the Czech Republic it is not customary to return dogs to the place of capture after treatment). Only 9 cats were returned to the original environment, representing 2.1% of the entire monitored cat population. The captured individuals are often not able to adapt to the new environment of the shelter, so a suitable solution for them in terms of welfare is return them to their original habitat.

From the point of view of the impact of sex on the length of stay in the shelter until adoption, males were more preferred in cats and females in the case of dogs. However, more feline females and canine males were adopted, which was related to their higher numbers in the shelter. According to Kubesova et al., [30] female cats stay shorter in the shelter than male cats (female cats’ median 43 days and male cats’ median 50 days) and their adoption rate is higher (55.8%), which is confirmed in the study by Onodera et al., [6], 55.2% of adopters preferred a female cat to a male cat. Opposite results-meaning an the increased preference of males-was recorded by American authors [15,17,45]. The preference of females in cats may be related to their greater prevalence in shelters [30]. Owners get rid of pregnant cats in order to avoid taking care of their offspring.

Zak et al., [3] reported, similarly to our results, the shorter length of stay in the shelter in female dogs than male dogs, which other foreign authors confirm in their studies [8,37,46]. An opposite result (with the increased male canine preference) can be found in a Brazilian study by Soto et al. [47]. Brown et al., [38] did not find any difference in the preference of males or females. There may be a number of reasons for the adoption preference of females in both cats and dogs. One of them is the calmer nature of the female sex [37]. Unlike females, male dogs tend to wander and show higher aggressiveness - due to these attributes, potential adopters may consider this sex less attractive [47]. The disadvantage of adopting females may be the potential risk of unwanted offspring. In a study by Soto et al., [47] adopters said that male dogs, unlike females, require less attention. It has been found that neutered animals are even more likely to be adopted than unneutered ones [48]. However, our results show that cats despite being routinely neutered in the Czech shelters have a longer median LOS until adoption than dogs that are adopted unneutered.

After assessing the age of cats and dogs in the shelter, kittens and puppies were found to remain in the shelter for the shortest time. In contrast, the oldest category of animals stayed in the shelter for the longest time. Furthermore, old cats stayed in the shelter longer than old dogs, despite fewer old cats than old dogs being admitted to the shelter (6.5% old cats vs. 23.7% old dogs). Foreign [6,17] as well as Czech authors found that kittens and young cats are the most favored group [30]. The adoption rate of a certain age category reflects the age of the animals on admission. Puppies and young dogs stay in the shelter for the shortest time [3,38,45], In contrast, the oldest dogs stay there for the longest time [3,49]. The chance of adoption decreases with the rising age [45]. The preference of young age categories may be related to the nice appearance and activity of the animals. This factor plays an important role in contributing to faster adoption [20]. The advantage of adopting a young animal lies in the assumption of good health and a high level of adaptability to the new environment. In contrast, older animals often suffer from health problems [50], which may pose a disadvantage for the new adopters in the form of higher financial costs for care. According to Clancy and Rowan [51], this group of animals in shelters suffers from a lack of attention from potential adopters.

Statistical testing found that purebred status does not affect the length of stay in the shelter until adoption, either in dogs or in cats. Cats and dogs that showed signs of belonging to a particular breed did not spend significantly less time in the shelter than crossbreds. However, the number of adopted crossbreds was higher than the number of adopted animals with purebred features, which was due to their higher numbers in shelters. The reason for adopting a purebred cat or dog may be a certain level expectations from people of the exterior of the animals-the appearance is one of the main criteria for selecting an animal from a shelter [2] and it is often preferred by the potential adopter, even over the animal’s behavior and temperament [45]. In general, crossbreds are more frequently taken to shelters [34], however, preference is given to animals showing signs of particular breeds, which remain in shelters for shorter lengths [17,33,40,45]. The purebred animals possess a higher value for the public, also due to their unconventional appearance and “uniqueness”.

## 5. Conclusions

In conclusion, the factors affecting the length of stay in the shelter until adoption, which were monitored in this study, differ in many aspects between cats and dogs. In terms of practical implications, the most significant difference seems to be the considerably different length of stay of a dog and cat in the shelter until adoption; cats seem to have a worse position for adoption, due to the longer time spent in the shelter compared to dogs. This finding reflects the current situation of the relationship between people and the two most commonly kept animal species (dogs and cats) in the Czech Republic (therefore, studies with a similar target in other countries in the world could produce different results). Czech shelters and other temporary care facilities for stray and abandoned animals should perceive this as a key factor; with targeted efforts and education of the public, it is possible to mitigate the negative effects of favoring a certain category of animals over others.

## Figures and Tables

**Table 1:** Population of evaluated cats and dogs; number of animals and the median LOS in variables of the way of outcome.

Way of Outcome	No.	%	LOS (Median of Days)
Adopted		*p* < 0.001 ^#^	*p* < 0.001 ^&^
Cats	367	87.6	53
Dogs	1073	41.6	27
Having died		*p* < 0,05 ^#^	*p* > 0.05 ^&^
Cats	17	4.1	25
Dogs	56	2.2	13
Euthanized		*p* > 0.05 ^#^	*p <* 0.001 ^&^
Cats	2	0.5	7
Dogs	32	1.2	79
Reclaimed		*p* < 0.001 ^#^	*p* < 0.001 ^&^
Cats	10	2.4	4
Dogs	1343	52.1	0
Captured and released	-		-
Cats	9	2.1	40
Dogs	-	-	-
Unadopted		*p* > 0.05 ^#^	-
Cats	14	3.3	-
Dogs	76	2.9	-

^#^ Chi-square test; ^&^ Kruskal-Wallis ANOVA.
